# Risk of prolonged postoperative opioid use after elective shoulder replacement: a nationwide cohort study of 5,660 patients from the Danish Shoulder Arthroplasty Registry

**DOI:** 10.2340/17453674.2024.41090

**Published:** 2024-08-15

**Authors:** Alexander Scheller MADRID, Jeppe Vejlgaard RASMUSSEN

**Affiliations:** Department of Orthopaedic Surgery, Herlev and Gentofte Hospital, Copenhagen University Hospital, Denmark

## Abstract

**Background and purpose:**

Several studies from the United States report an increased risk of prolonged opioid use after shoulder replacement. We aimed to determine the incidence and risk factors of prolonged opioid use after elective shoulder replacement in a nationwide Danish population.

**Methods:**

All primary elective shoulder arthroplasties reported to the Danish Shoulder Arthroplasty Registry (DSR) from 2004 to 2020 were screened for eligibility. Data on potential risk factors was retrieved from the DSR and the National Danish Patient Registry while data on medication was retrieved from the Danish National Health Service Prescription Database. Prolonged opioid use was defined as 1 or more dispensed prescriptions on and 90 days after date of surgery (Q1) and subsequently 1 or more dispensed prescriptions 91–180 days after surgery (Q2). Preoperative opioid use was defined as 1 or more dispensed prescriptions 90 days before surgery. Logistic regression models were used to estimate risk factors for prolonged opioid use.

**Results:**

We included 5,660 patients. Postoperatively 1,584 (28%) patients were dispensed 1 or more prescriptions in Q1 and Q2 and were classified as prolonged opioid users. Among the 2,037 preoperative opioid users and the 3,623 non-opioid users, 1,201 (59%) and 383 (11%) respectively were classified as prolonged users. Preoperative opioid use, female sex, alcohol abuse, previous surgery, high Charlson Comorbidity index, and preoperative use of either antidepressants, antipsychotics, or benzodiazepines were associated with increased risk of prolonged opioid use.

**Conclusion:**

The incidence of prolonged opioid use was 28%. Preoperative use of opioids was the strongest risk factor for prolonged opioid use, but several other risk factors were identified for prolonged opioid use.

Interest in opioid use after orthopedic procedures is increasing, as focus is directed towards the association of surgery and development of chronic opioid use [1-4]. Short-term use of analgesics including paracetamol, non-steroidal anti-inflammatory drugs (NSAID), and opioids is the keystone in alleviating post-procedural pain. The use of opioids comes with a risk of dependence, misuse, and diversion, and utilization has increased dramatically worldwide as have the number of deaths related to opioid overdoses [5,6]. Presently, according to the Centers for Disease Control and Prevention (CDC), most opioid-related deaths are attributed to illicit synthetic opioid use, particularly fentanyl [7]. However, prescription opioids might precede illicit drug use [8]. Orthopedic surgeons are one of the main prescribers of opioids and orthopedic surgery show some of the highest rates of postoperative chronic opioid use [9-11]. This emphasizes the importance of understanding postoperative opioid usage.

Several studies have reported high prevalence of prolonged opioid usage after shoulder arthroplasty, varying from 15–30% [1,3,12,13]. However, these studies used varying definitions of preoperative and postoperative opioid usage thus hindering comparison. Additionally, all studies originate from the United States, therefore potentially lacking generalizability [8,14]. Age, female sex, preoperative use of opioids, depression, anxiety, and a history of alcohol abuse have all been identified as risk factors for prolonged opioid use [1,3].

Our primary aim was to report the incidence of patients with prolonged opioid use after elective shoulder replacement, and secondarily to identify risk factors for prolonged opioid use in a nationwide Danish population.

## Methods

### Data sources

The Danish Shoulder Arthroplasty Registry (DSR) has previously been used in determining patient-reported outcomes and arthroplasty survival rates [15,16]. The DSR is a nationwide registry established in 2004. After 2006 it was mandatory for all Danish public and private hospitals performing shoulder replacement to report to the DSR. Data related to the patient and the operation is reported by the surgeon at the time of the operation. The completeness of reporting is 94% and there is high accuracy of reporting age, operated side, sex, indication for surgery, previous surgery, and type of arthroplasty [17].

Data on drug use was collected from the Danish National Health Service Prescription Database, which includes information on all prescriptions for dispensing by community pharmacies according to the Anatomical Therapeutic Chemical classification system (ATC codes). The database includes name and brand of the drug, quantity, formulation, date of refill, the prescribing physicians, and the dispensing pharmacy. Data has been collected since 1995 [18].

The Danish National Patient registry was used to obtain information on all physician-assigned diagnoses from emergency departments, outpatient clinics, and inpatient admissions to all hospitals in Denmark. Diagnoses are coded according to the International Classification of Diseases; Tenth Revision (ICD-10) [19]

### Study population

All primary elective shoulder arthroplasties reported to the DSR from 2004 to 2020 were screened for eligibility. Only primary shoulder arthroplasties for osteoarthrosis, cuff tear arthropathy, and humeral head necrosis were included.

### Definition of covariates

Opioid prescriptions (ATC N02A) dispensed from community pharmacies 90 days before and 365 days after surgery were obtained through the Danish National Health Service Prescription Database. We did not have data on opioids given to the patient by the hospital during admission or on discharge, or prescribed but not dispensed opioid prescription.

Patients with diabetes mellitus were defined as using antidiabetic medication (ATC A10) or having a preoperative diagnosis of diabetes (ICD10 E08–E14) before surgery. Similarly, preoperative diagnosis of alcohol abuse (ICD10 F10.1–F10.9), chronic pulmonary disease (ICD10 J44), and preoperative use of antipsychotic medication (ATC N05A), benzodiazepines (ATC N05BA and N05CD–N05CF), and antidepressants (ATC N06A) was collected. The Charlson Comorbidity index was based on diagnoses in the Danish National Patient registry, which were obtained from the DSR.

### Pre- and postoperative opioid usage

Preoperative opioid use was defined as 1 or more dispensed prescription of opioids within 90 days before surgery. Postoperative opioid use was defined as dispensed prescription opioids on or after day of surgery. For statistical analysis opioid utilization was dichotomized and labelled as prolonged or not prolonged opioid use. Prolonged opioid use was defined as 1 or more dispensed opioid prescriptions from date of surgery until 90 days after day of surgery and subsequently 1 or more prescription within 91 to 180 days after surgery. This definition is in accordance with CDC’s definition of chronic pain (lasting > 3 months or past the time of normal tissue healing) [20] and the ICD-11 definition of chronic postsurgical pain [21]. The definition has previously been used in a similar study [22]. Furthermore, early recognition of inappropriate opioid use or diagnosis of chronic postsurgical pain is more viable in a clinical setting. Therefore, we did not include opioid dispensing in the third and fourth quartile in our statistical analysis. However, for between-study comparison proportions of patient dispensing within each quarter for the first postsurgical year is presented.

### Statistics

Descriptive statistics including frequencies, proportions, means, and standard deviations (SD) are presented for baseline characteristics for both preoperative opioid users and preoperative opioid non-users. Continuous variables followed the assumption of normal distribution.

Univariate and multivariable logistic regression analysis was performed to estimate risk of prolonged opioid use. Preoperative opioid use, age (grouped as below or above 65 years of age, based on previous studies [1,12]), sex, diabetes mellitus, alcohol abuse, chronic pulmonary disease, Charlson Comorbidity Index, preoperative use of antidepressants, antipsychotics, or benzodiazepines, type of arthroplasty, and year of surgery were included in the model. Multivariable logistic regression analysis stratified by preoperative opioid use was performed. Preoperative opioid users were additionally adjusted by the number of opioid prescriptions dispensed within 90 days before surgery. Estimates were given with odds ratio (OR) and 95% confidence intervals (CI). No major violation of assumptions for logistic regression was observed.

Postoperative opioid dispensing for the first postoperative year is presented as frequencies for each quartile postoperatively for both preoperative opioid non-users and preoperative opioid users. Additionally, for each opioid prescription, the oral morphine equivalent (OMEQ) was estimated using standardized conversion factors (Table 1, see Appendix). Cumulative postoperative OMEQs in the first 7, 30, 60, 90, 120, 150, and 180 days after surgery were then calculated for preoperative opioid users and preoperative opioid non-users. Median and interquartile range are used due to skewness of data.

**Table 1:** Conversion factor for calculating oral morphine equivalence (OMEQ)

Opioid	Conversion factor
Morphine	1.0
Hydromorphone	5.0
Nicomorphine	1.0
Oxycodone	1.5
Ketobemidone (Ketogan)	2.0
Pethidine	0.1
Fentanyl	7.2 (transdermal)**^a^**
Dextropropoxyphene**^b^**	0.2
Codeine	0.15
Tradolan	0.2
Tapentadol	0.4
Buprenorphine **^c^**	12.6 (transdermal)**^c^**

a Transdermal fentanyl has a daily conversion factor of 2.4 and is normally applied for 3 days. Therefore, OMEQ was calculated as number of patches x 2.4 x 3 = 7.2.

b Dextropropoxyphene discontinued in 2010 due to increased risk of fatal poisoning.

c Buprenorphine has a daily conversion factor of 1.8 and is normally applied for 7 days. Therefore, OMEQ was calculated as number of patches x 1.8 x 7.

All statistical analyses were performed in Stata version 17.0 (StatCorp LLC, College Station, TX, USA).

### Ethics, funding, data sharing, and disclosures

Approval by the local ethics committee was not required for registry studies. The study was approved by the Danish Data Protection Agency (registration number: P-2020-586). Data is available from the Danish registries given that approval from the registries can be provided. A grant was received from the Danish Society for Shoulder and Elbow Surgery for data acquisition and the Danish Society for Shoulder and Elbow Surgery was not otherwise involved in this study. Authors declare no conflict of interests. Complete disclosure of interest forms according to ICMJE are available on the article page, doi: 10.2340/17453674.2024.41090

## Results

### Preoperative baseline characteristics

**Figure 1:**
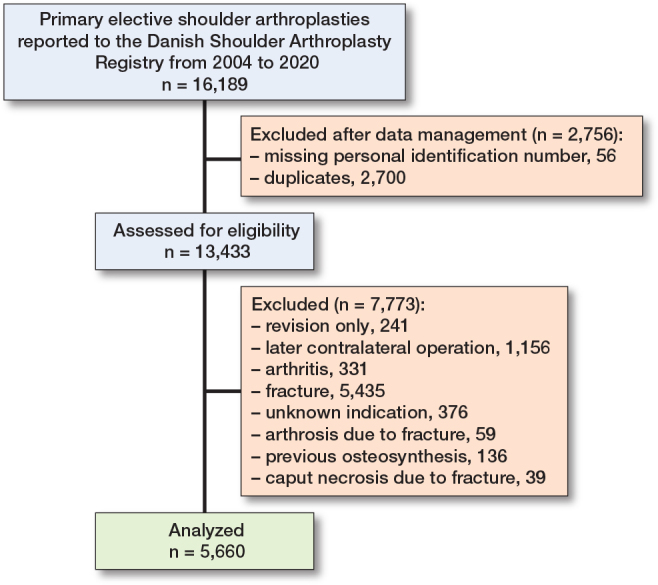
Flowchart.

**Figure 2:**
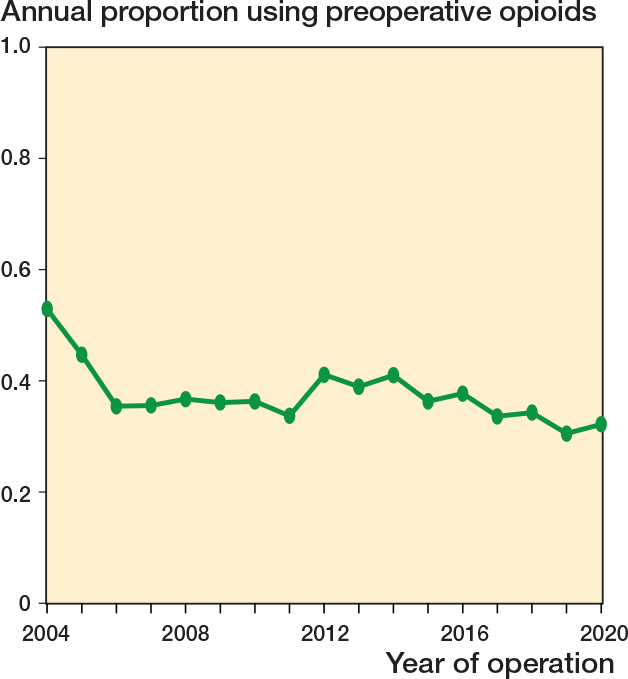
Proportion of patients using opioids within 90 days preoperatively as a function of year of operation.

Patients with acute fracture, fracture sequelae, and arthritis were excluded. Additionally, patients with later contralateral shoulder arthroplasty were excluded (n = 1,144) (Figure 1). We thus included 5,660 patients of whom 2,037 (36%) were dispensed at least 1 prescription for an opioid within 90 days before surgery (Table 2). Preoperative opioid users were more likely female, diabetics, and users of antidepressants, antipsychotic, or hypnotics. For preoperative opioid users, 691 (34%) patients were dispensed 1 opioid prescription, 1,009 (50%) patients were dispensed 2–4 opioid prescriptions and 337 (17%) patients were dispensed ≥ 5 opioid prescriptions within 90 days before surgery (Table 2). The proportion of preoperative opioid users remained stable during the study period (Figure 2, see Appendix).

**Table 2:** Preoperative baseline characteristics. Values are count (%) unless otherwise specified

Factor	No preoperative opioid use (n = 3,623)	Preoperative opioid user (n = 2,037)
Age, mean (SD)	70 (10)	70 (11)
Male sex	1,549 (43)	698 (34)
Diabetes	374 (10)	304 (15)
Alcohol abuse	86 (2.4)	89 (4.4)
Chronic pulmonary disease	23 (0.6)	36 (1.8)
Previous surgery	918 (25)	600 (29)
Charlson Comorbidity index		
0	2,145 (59)	909 (45)
1–2	1,128 (31)	756 (37)
≥ 3	350 (10)	372 (18)
Preoperative drug use		
Antidepressants	479 (13)	563 (28)
Antipsychotics	220 (6.1)	341 (17)
Hypnotics	341 (9.4)	431 (21)
Preoperative opioid prescriptions		
1	N/A	691 (34)
2–4	N/A	1,009 (50)
≥ 5	N/A	337 (17)

### Postoperative opioid dispensing

For preoperative opioid users, 1,859 (91%) patients were dispensed a prescription opioid in the first quarter after surgery. Dispensing of an opioid prescription decreased over time with 1,226 (60%) patients in Q2 (day 90–180), 1,168 (57%) patients in Q3, and 1,148 (56%) patients in Q4. In the group of patients who did not use opioids preoperatively the number of patients who received an opioid prescription was 2,583 (72%) in Q1, and 433 (12%) in Q2, 370 (10%) in Q3, and 392 (11%) in Q4.

### Risk factors of prolonged opioid use

**Figure 3:**
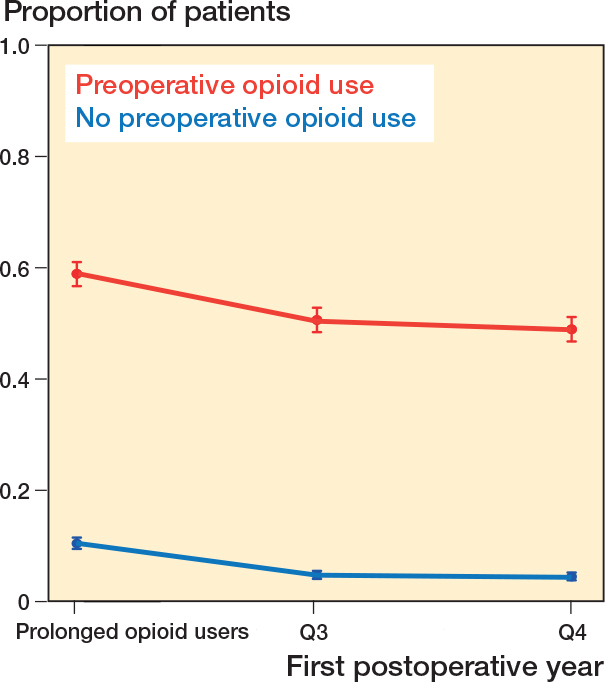
Proportion of patients defined as prolonged opioid users stratified by preoperative use and proportion of patients with opioid use in the subsequent quarters (Q) of the first postoperative year. Proportion and 95% confidence interval are shown.

1,584 (28%) patients were dispensed 1 or more prescriptions in Q1 and Q2 and were classified as prolonged opioid users. Among the 2,037 preoperative opioid users and the 3,623 non-opioid users, 1,201 (59%) and 383 (11%) were classified as prolonged users. Continued quarterly use declined for prolonged opioid users (Figure 3, see Appendix).

In a univariate logistic regression model, several risk factors for prolonged opioid use were identified. Preoperative opioid use was strongly associated with an unadjusted OR of 12.2 (CI 10.6–13.9) and a multivariable adjusted OR of 10.1 (8.8–11.7) (Table 3).

**Table 3:** Risk of opioid use beyond 90 days after shoulder arthroplasty. Values are count (%) unless otherwise specified

Factor	Not prolonged opioid use (n = 4,076)	Prolonged opioid use (n = 1,584)	OR (CI) ^a^	P	adjOR (CI) ^b^	P
Age						
< 65 years	1,144 (28)	449 (28)	Ref.		Ref.	
≥ 65 years	2,932 (72)	1,135 (72)	0.99 (0.87–1.12)	0.8	0.88 (0.74–1.05)	0.2
Male sex	1,742 (43)	505 (32)	0.63 (0.55–0.71)	< 0.01	0.71 (0.61–0.83)	< 0.01
Diabetes	436 (11)	242 (15)	1.51 (1.27–1.78)	< 0.01	1.13 (0.91–1.41)	0.3
Alcohol	84 (2.1)	91 (5.7)	2.90 (2.14–3.92)	< 0.01	2.12 (1.44–3.13)	< 0.01
Chronic pulmonary disease	24 (0.6)	35 (2.2)	3.81 (2.26–6.43)	< 0.01	1.77 (0.89–3.50)	0.1
Charlson Comorbidity Index						
0	2,357 (58)	697 (44)	Ref.		Ref.	
1–2	1,288 (32)	596 (38)	1.56 (1.36–1.78)	< 0.01	1.22 (1.04–1.43)	0.01
≥ 3	431 (11)	291 (18)	2.28 (1.92–2.71)	< 0.01	1.37 (1.09–1.72)	< 0.01
Previous surgery	1,016 (25)	502 (32)	1.40 (1.23–1.59)	< 0.01	1.35 (1.15–1.59)	< 0.01
Preoperative drug use						
Antidepressants	563 (14)	479 (30)	2.70 (2.35–3.11)	< 0.01	1.54 (1.29–1.82)	< 0.01
Antipsychotics	258 (6.3)	303 (19)	3.50 (2.93–4.18)	< 0.01	1.74 (1.39–2.17)	< 0.01
Benzodiazepines	407 (10)	365 (23)	2.70 (2.31–3.15)	< 0.01	1.62 (1.34–1.96)	< 0.01
Preoperative opioids	836 (20)	1,201 (76)	12.2 (10.6–13.9)	< 0.01	10.1 (8.8–11.7)	< 0.01
Year of operation	N/A	N/A	0.97 (0.96–0.99)	< 0.01	1.00 (0.98–1.02)	0.7
Arthroplasty implant						
Total	1,322 (33)	383 (24)	Ref.		Ref.	
Reverse	1,362 (34)	626 (40)	1.58 (1.37–1.84)	< 0.01	1.25 (1.05–1.50)	0.02
Hemi	1,340 (33)	556 (35)	1.43 (1.23–1.67)	< 0.01	1.25 (1.01–1.55)	0.06

a Univariate logistic regression analysis with 95% confidence interval (CI)

b adjOR = multivariable adjusted odds ratio with CI.

Moreover, female sex, alcohol abuse, previous surgery, high Charlson Comorbidity index, and use of antidepressants, antipsychotics, and benzodiazepines were identified as risk factors for prolonged opioid use (Table 4).

**Table 4:** Risk factors of prolonged postoperative opioid use for preoperative opioid user and non-users

Factor	No preoperative opioid user (n = 3,623)		Preoperative opioid use (n = 2,037)	
	adjOR (CI)	P	adjOR (CI)	P
Age				
< 65	Ref.		Ref.	
≥ 65	0.92 (0.71–1.21)	0.6	0.89 (0.70–1.13)	0.3
Male sex	0.77 (0.61–0.97)	0.03	0.67 (0.54–0.83)	< 0.01
Diabetes	1.29 (0.91–1.81)	0.2	1.01 (0.75–1.36)	0.9
Alcohol abuse	2.56 (1.51–4.35)	< 0.01	1.56 (0.88–2.76)	0.1
Chronic pulmonary disease	0.83 (0.24–2.91)	0.8	3.19 (1.03–9.87)	0.04
Charlson Comorbidity Index				
0	Ref.		Ref.	
1–2	1.20 (0.94–1.53)	0.1	1.13 (0.91–1.41)	0.3
≥ 3	1.33 (0.92–1.94)	0.1	1.28 (0.95–1.74)	0.1
Previous surgery	1.51 (1.18–1.93)	< 0.01	1.28 (1.02–1.61)	0.03
Preoperative drug use				
Antidepressants	1.38 (1.04–1.84)	0.03	1.41 (1.12–1.79)	< 0.01
Antipsychotics	1.67 (1.15–2.43)	< 0.01	1.55 (1.16–2.09)	< 0.01
Benzodiazepines	1.79 (1.31–2.44)	< 0.01	1.38 (1.07–1.77)	0.01
Preoperative opioid prescriptions				
1			Ref.	
2–4			2.92 (2.37–3.58)	< 0.01
≥ 5			7.44 (5.26–10.56)	< 0.01
Arthroplasty implant				
Total	Ref.		Ref.	
Reverse	1.24 (0.94–1.64)	0.1	1.31 (1.02–1.68)	0.03
Hemi	1.20 (0.86–1.67)	0.3	1.34 (1.00–1.79)	0.05

Multivariable logistic regression analyses for risk of prolonged opioid use.

adjOR = multivariable adjusted odds ratio. CI = 95% confidence interval.

**Figure 4:**
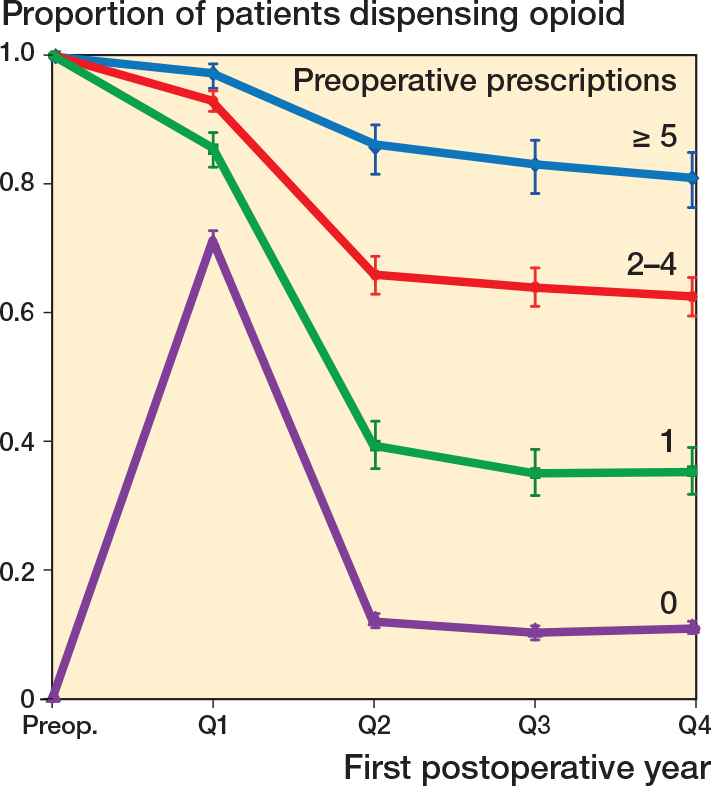
Proportions of patients dispensed opioids for each quarter in the first postoperative year, as function of preoperative dispensed opioid prescriptions within 90 days before date of surgery. Proportion and 95% confidence interval is presented.

For the group of preoperative opioid users, the OR for prolonged opioid use was 2.9 (CI 2.4–3.6) for patients who were dispensed 2–4 prescriptions and 7.4 (CI 5.3–10.6) for patients who were dispensed > 5 opioid prescriptions with patients who were dispensed 1 prescription as reference (Table 4). Accordingly, the proportions of patients who were dispensed medications within each quarter as a function number of dispensed opioid prescriptions showed a strong association graphically (Figure 4).

### Oral morphine equivalent

**Figure 5:**
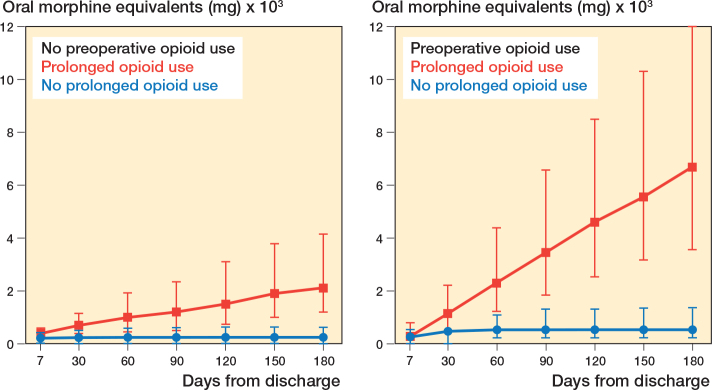
Postoperative oral morphine equivalents (OMEQ) represented as median and interquartile range at 7, 30, 60, 90, 120, 150, and 180 days from the date of surgery. Data is shown separately for preoperative opioid users and non-users for both prolonged opioid users and not prolonged opioid users.

For preoperative opioid users and non-users without prolonged opioid use, the median cumulated OMEQ stabilized within 30 days. For patients defined as prolonged opioid users we observed a linear increase in median cumulated OMEQ in the first 180 days postoperatively for both preoperative opioid users and non-users (Figure 5).

## Discussion

We aimed to determine the incidence and risk factors of prolonged opioid use after elective shoulder replacement in a nationwide Danish population. This is the first study outside the United States reporting opioid usage before and after shoulder arthroplasty. 36% of all patients with shoulder replacement received at least 1 opioid prescription 90 days before surgery. 59% of opioid users and 11% of non-opioid users had prolonged opioid use postoperatively. Preoperative opioid use, female sex, alcohol abuse, high Charlson Comorbidity Index, and preoperative use of antidepressants, antipsychotics, or benzodiazepines were identified as risk factors for prolonged opioid use.

Previous studies have reported preoperative and postoperative opioid usage after shoulder arthroplasty that is comparable to our findings. Khazi et al. used the Humana claims database on 12,038 patients who were treated with total shoulder arthroplasty and found that 43% fulfilled an opioid prescription within 3 months preoperatively. They observed that 31% of preoperative opioid users and 3.1% of non-opioid users were fulfilling prescriptions of opioids in the 12th month postoperatively [1]. Leroux et al. used the same database and included preoperative opioid non-users for various types of elective shoulder procedures; they reported that 14% and 20% were using opioids more than 180 days after total shoulder arthroplasty and hemiarthroplasty respectively [3]. Our study showed similar opioid utilization patterns; however, direct comparison is hindered by the varying definitions of preoperative and postoperative opioid utilization. For preoperative opioid users we observed a strong association between number of prescriptions and prolonged opioid use. This is supported by Rao et al., who observed a 10 times higher risk of using opioids in Q4 after surgery when patients received ≥ 5 opioid prescriptions compared with patients who did not receive preoperative opioids as the reference [12]. Khazi et al. showed that opioid prescription 1–3 months before surgery was associated with a 10 times increased risk of opioid use 12 months postoperatively compared with patients with no preoperative opioid use. Patients who received an opioid prescription less than 1 month before surgery had an odds ratio of only 2.5 [1].

Our study implies that there is a large proportion of patients receiving opioids preoperatively. Despite increased awareness of the harms of opioids, we did not observe a decrease in preoperative opioid usage between 2004 and 2020. One explanation could be that although opioids have received growing attention, no formalized national policies or restrictions have been instituted in Denmark. The number of patients with prolonged opioid use after shoulder replacement resembles those observed from studies originating from the United States. It is important to keep in mind that direct comparison is not possible due to different definitions of prolonged or chronic opioid use. Our definition of prolonged opioid use was based on the definition proposed by the CDC and ICD-11. Though opioids remain one of the cornerstones in postoperative pain management, newer studies report promising results in minimizing opioid consumption. Jones et al. demonstrated similar pain scores using an opioid-sparing protocol, resulting in a fourfold reduction in opioid consumption and earlier opioid discontinuation after shoulder arthroplasty [23]. Jolisaints et al. reported equal or better pain scores using an opioid-free postoperative pain protocol [24]. Likewise national policies restricting opioid prescribing lead to significant reductions in postoperative opioid prescriptions [25]. These studies are mainly focused on patients without preoperative opioid use although preoperative opioid use has consistently been shown to be one of the most important risk factors for prolonged use. The literature on managing postoperative pain for preoperative opioid users is limited, with small studies recommending preoperative weaning or personalized opioid tapering [26,27].

### Strengths

This study includes a high number of participants included from all Danish hospitals performing shoulder replacement, with high completeness of reporting and follow-up, and with national registration of all dispensed opioids in Denmark.

### Limitations

First, due to this study’s observational nature we are unable to give etiological reasons for preoperative and prolonged postoperative opioid use, as this could be due to persistent postoperative pain, physician prescribing patterns or, maybe most importantly, concurrent musculoskeletal pain or trauma. We excluded patients with later contralateral shoulder arthroplasty, not only to avoid violation of the assumption of independence in our risk estimates but also to diminish the risk of a painful contralateral shoulder being the reason for prolonged opioid use. Nevertheless, our findings are probably overestimated. Another limitation is the possible variation in dispensed and consumed opioids and the lack of information on possible hospital supplied opioids on discharge. Due to missing information on daily dose and duration for each opioid prescription, we are unable to give more precise estimates of prolonged opioid use than last dispensed opioid prescription. Lastly, the study consists of only Danish patients, and the Danish healthcare system is free and fully funded through taxes, which could reduce generalizability. Nonetheless, our results are comparable to opioid utilization reported from the United States.

### Conclusion

We observed that 28% of patients undergoing elective shoulder surgery were prolonged opioid users. Preoperative opioid use was the strongest risk factor for prolonged opioid use. Additionally female sex, alcohol abuse, previous surgery, high Charlson Comorbidity Index, and use of antidepressants, antipsychotics, and benzodiazepines were identified as risk factors for prolonged opioid use after elective shoulder replacement.

### Perspective

Surgeons should be aware of the identified risk factors in pain management after shoulder replacement. An opioid-limiting, multimodal, and individualized pain management program might decrease prolonged opioid use. There is a need for studies investigating optimized strategies for managing postoperative pain in patients with and without preoperative opioid use.

## Notes

ASM: Study setup, data acquisition, data analysis and statistics, writing. JVR: Study setup, data acquisition, writing, and proofreading.

The authors are grateful to the Danish Society for Shoulder and Elbow Surgery for supporting them in the completion of this study.

Handling co-editors: Taco Gosens

*Acta* thanks Carl-Olav Stiller and other anonymous reviewers for help with peer review of this manuscript.
